# On the Relationship between Contact Resistance and Load Force for Electrode Materials with Rough Surfaces

**DOI:** 10.3390/ma15165667

**Published:** 2022-08-18

**Authors:** Chao Zhang, Wanbin Ren, Xiaoyu Liao

**Affiliations:** 1 School of Electrical Engineering and Automation, Harbin Institute of Technology, Harbin 150001, China; 2 School of Instrumentation Science and Engineering, Harbin Institute of Technology, Harbin 150001, China; 3 Xi’an Aeronautics Computing Technique Research Institute, Aviation Industry Corporation of China, Xi’an 710065, China

**Keywords:** contact resistance, load force, electrode material, contact *a*-spot, surface roughness

## Abstract

Higher contact resistance not only increases power consumption and temperature rise but also causes undesirable interconnectivity between electrode materials, which further influences the electrical lifespan and reliability of switching devices. However, relevant studies on the relationship between contact resistance and load force, and on the reduction of contact resistance by controlling the micro-structure of rough surfaces, especially for electrode materials with larger *S*_q_ (root mean square) values, are very limited. In this study, the contact resistance calculation method, based on classical Holm theory in combination with the elastic and plastic deformation, was reviewed. Then, typical curves of measured contact resistance and load force were analyzed and compared with the calculation results for smooth surfaces. Furthermore, experimental results for electrodes with bright and matt surfaces were compared. It was found that the average contact resistance of samples with matt surfaces was 0.162 mΩ for a load force of 5 N, which decreased by 18.52% compared to that of the bright surface. The standard deviation of the contact resistance greatly decreased to 0.008 mΩ for samples with matt surfaces, which indicated that the matt electrode surface could effectively produce low and stable contact resistance. In addition, the influences of the numbers and sizes of contact *a*-spots on the relationship between contact resistance and load force were investigated. It was found that denser asperities with smaller curvature radii for the matt surface were beneficial for lower contact resistance, even for the electrode material with larger *S*_q_ values. Finally, an empirical model of the contact resistance with error bands based on the experimental results was established and verified.

## 1. Introduction

Electrode materials are widely used in many electrical and electronic components, such as electromechanical devices, connectors, thin-film devices, micro-electromechanical systems and switch gears, to conduct load currents [1,2,3,4,5]. All surfaces of electrode materials are microscopically rough, even the macroscopically polished surfaces [6]. The electrical contact resistance across an interface is an important quantity and is closely related to the physical properties of electrode materials, such as the contact structure, surface state, load force and load current. A low and stable contact resistance can decrease the temperature rise and power consumption and, further, improve the electrical lifespan and reliability of switching devices [7,8].

The theory of contact resistance has long been a hot academic issue. In the 1940s, Holm [9] first presented the theoretical calculation method for the constriction resistance of a single circular *a*-spot based on the electrical resistivity of electrode materials and the contact radius of the contact *a*-spot. Combining the Hertz contact with Tabor theory, it was found that the constriction resistance is proportional to the contact load force, with −1/3 and −1/2 exponential powers for the elastic and plastic deformation. Aiming to calculate the total conductance of rough surfaces, Greenwood and Williamson [10] further proposed a contact mechanics model by assuming the asperity heights follow a Gaussian distribution. Subsequently, many researchers have devoted much effort to establishing a calculation model for contact resistance by considering surface roughness [11,12,13,14]. Riba et al. [15] proposed a simple FEM-based method to estimate the constriction resistance and found that, when the contact pressure is beyond a certain threshold, the constriction resistance is almost independent of the apparent area of contact for copper samples with *S*_q_ values of 0.25 μm. Liu et al. [16] calculated the characteristics of the electrical contact between a hemisphere and rough Au/CNT surfaces with a parameter *S*_q_ ranging from 0.1–1 μm and found that the contact resistance would increase with surface roughness.

In addition, investigations using experimental methods of the relationship between the electrical contact resistance and the contact load force, considering the surface topography of electrode materials, have attracted much attention. Hernot et al. [17] reported that the discrepancy between the theoretical and experimental results for the contact resistance of bulk metals (Ag, Au, Pd, Ni), especially with low contact force, results from ignoring the surface roughness of the electrode materials. Tamai et al. [18] found that the contact resistance decreases singularly with an exponential power of −2, which deviates significantly from the classical value of −0.5 for plastic deformation. This could be attributed to the probe tip sinking into the soft tin surface, due to the separation and accumulation of the crystal grains, and the periphery of the contact area severely sliding against the surface side of the probe. Kwon et al. [19] showed that the relationship between contact resistance and load force for gold thin-film contacts with *S*_q_ (root mean square) values of 0.0036 μm and 0.0069 μm could be divided into three regions, and the power exponent of the load force changed from −0.45 in the stable contact regime to −0.08 in the saturated regime. The experimental results also indicated that the thicker gold film could effectively reduce the contact resistance, even for a larger surface roughness. The above investigations indicate that the relationship between contact resistance and load force obtained from experiments with practical electrode materials always deviates from the theoretical model due to the surface roughness. Although the law of variation of contact resistance for rough surfaces can be obtained directly from experiments, relevant studies on how to control contact resistance at the micro-scale in accordance with experimental results are very limited.

In the context of practical manufacture methods, the surface topography of electrode materials has a significant influence on contact resistance. There have also been considerable research efforts aimed at restraining the contact resistance to the utmost extent [20,21,22]. Fernandez et al. [23] developed a two-step annealing method by optimizing the contact surface morphology, and a lower contact resistance was achieved for ohmic contacts on n-GaN compared to the one-step annealing process. Recently, a novel surface modification method was introduced to manufacture electrode materials with matt features, which are different from traditional materials with bright features. Surprisingly, the associated contact resistance was reduced significantly. Unfortunately, the root reason for the reduction in the contact resistance in the matt electrode material has not been clarified.

The purpose of this study was to investigate the role of the surface topography of electrode materials on the contact resistance with the help of the relationship between electrical contact resistance and load force. Thus, the experimental results for silver-based alloy electrodes with bright and matt features were compared, and the influences of the numbers and sizes of contact *a*-spots on the contact resistance were interpreted explicitly. The corrected calculation model for contact resistance based on the classical Holm theory and experimental results was further established and verified.

## 2. Theoretical Background

**Figure 1:**
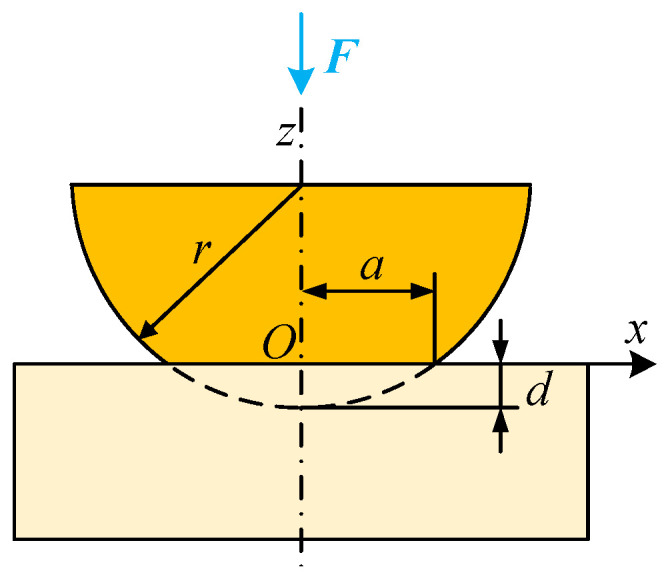
The schematic front view of the sphere–plane contact.

Traditionally, the mechanical contact between a cambered electrode and plane electrode was considered equivalent to the sphere–plane contact. The 2D contact model is described schematically in Figure 1. When the contact load force *F* is applied on the sphere with curvature radius *r*, the contact radius produced is *a*. The relationship between the load force *F* and contact radius *a* for elastic and plastic deformation can be written as [24]:

(1) $$F_{e}=\frac{4}{3}E^{*}\frac{a^{3}}{r}$$

(2) $$F_{p}=H\pi a^{2}$$

where E* = [(1 − *v*_1_^2^)/*E*_1_ + (1 − *v*_2_^2^)/*E*_2_]^−1^ is a constant consisting of the Young’s modulus and Poisson’s ratio of the involved materials and *H* is the hardness of the softer material.

The produced contact resistance presented by Holm can be expressed as [9]:

(3) $$R_{c}=\frac{\rho }{2a}$$

where *ρ* is the electrical resistivity of the electrode material. Substituting Equation (1) and Equation (2) into Equation (3), the relationships between contact resistance *R*_c_ and load force *F* for the elastic and plastic deformation of a sphere–plane contact are as follows [25]:

(4) $$R_{ce}=\rho {\left(\frac{E}{8rF}\right)}^{1/3}=K_{ce}F^{-0.33}$$

(5) $$R_{cp}=\rho {\left(\frac{\pi H}{4F}\right)}^{1/2}=K_{cp}F^{-0.5}$$

where *R_ce_* is the contact resistance for the elastic deformation situation with the coefficient $K_{\mathrm{ce}}=\frac{\rho }{2}{\left(\frac{E}{r}\right)}^{1/3}$, and *R_cp_* is the contact resistance for the plastic deformation situation with the coefficient $K_{\mathrm{cp}}=\frac{\rho }{2}{\left(\pi H\right)}^{1/2}$. It can be noted that the contact resistance and load force follow the power correlation according to Equations (4) and (5). The power exponents are −0.33 and −0.5, individually, for the elastic and plastic deformations.

## 3. Experimental Details

### 3.1. Test Rig

**Figure 2:**
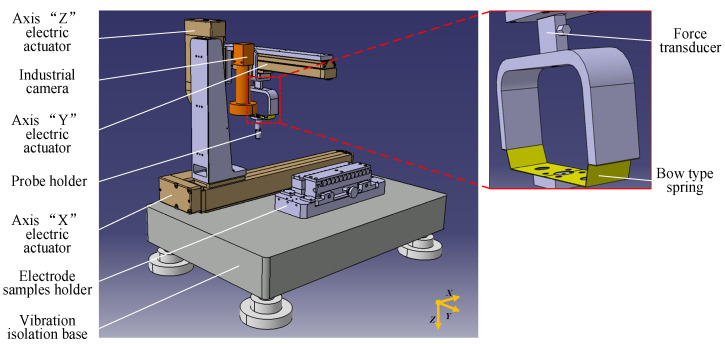
Three-dimensional (3D) model of the mechanical structure.

**Figure 3:**
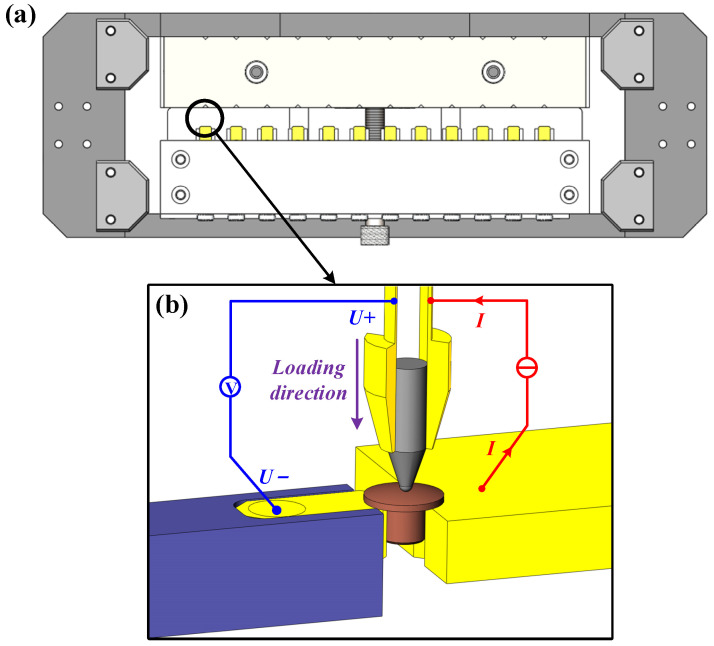
The schematic diagram of the clamping electrode sample. (**a**) The top view of the sample holder. (**b**) The four-wire configuration in one measurement workstation.

The test rig used in these experiments has been described in detail in [26]. We provide a brief summary here. The mechanical platform (shown in Figure 2) includes the 3D motion module, electrode clamping module and flexible loading module. Figure 3 schematically shows the four-wire measurement setup. The measurement resolution for contact resistance is 0.01 mΩ using the four-wire method.

### 3.2. Sample Description and Experimental Method

**Figure 4:**
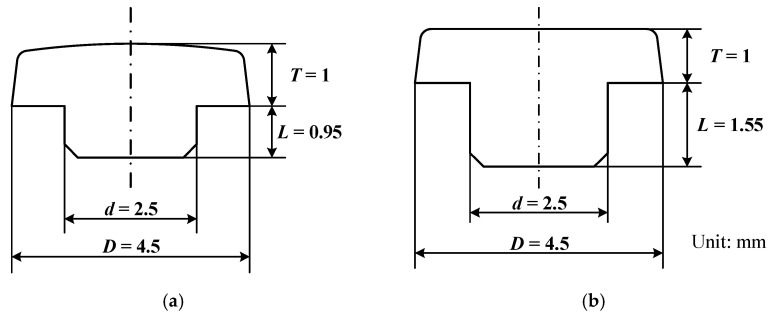
The sizes of the electrodes. (**a**) Movable sample. (**b**) Static sample.

**Figure 5:**
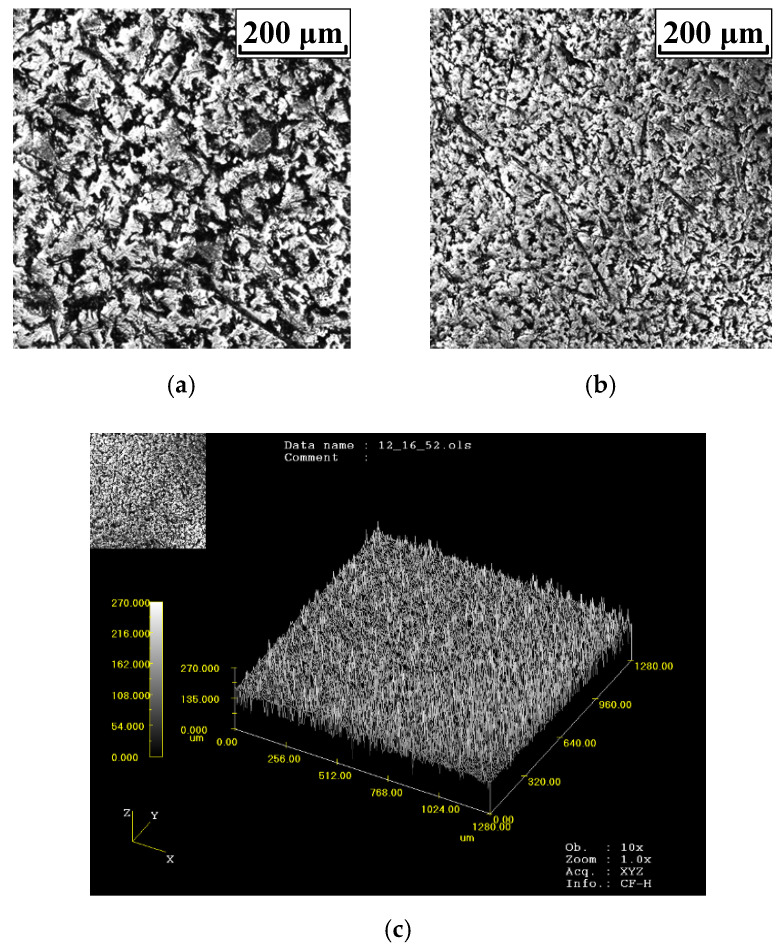

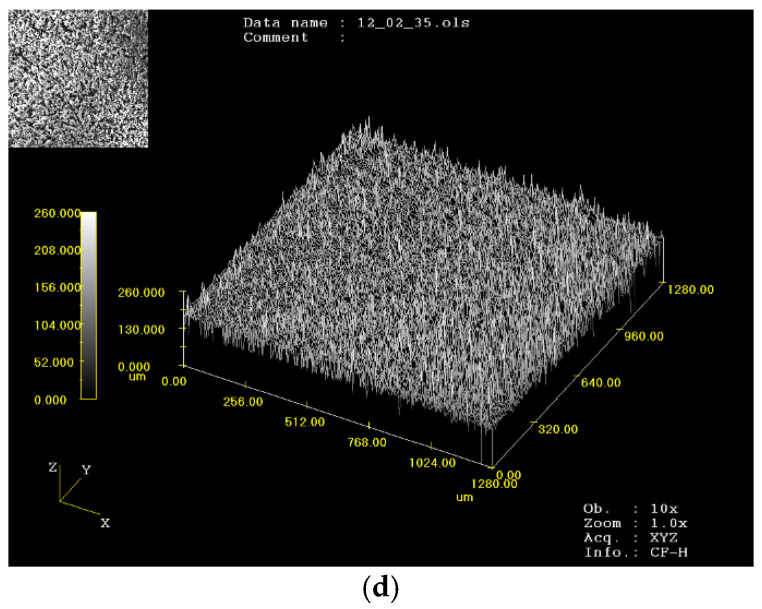
The laser confocal pictures for the bright and matt electrodes. (**a**) The surface topography of the bright electrode. (**b**) The surface topography of the matt electrode. (**c**) The surface wireframe of the bright electrode. (**d**) The surface wireframe of the matt electrode.

The selected electrode material was AgSnO_2_(12), and the applied additive was In_2_O_3_. The geometries of the movable sample and static sample are shown in Figure 4. Two representative AgSnO_2_(12) electrodes with different surface topographies were selected to investigate the electrical contact characteristics. The surface topographies of the bright electrode and the matt electrode with a scanning area of 1280 × 1280 μm^2^ are shown in Figure 5. The values for the measured parameter *S_q_* (root mean square height), which represents the height fluctuation of each point in the concerned region relative to the mean height, were 27.87 μm and 28.64 μm for the bright electrode and matt electrode, respectively. The measured hardness of the AgSnO_2_(12) material was 73.7HV(0.1 N) and the electrical resistivity was 2.35 × 10^−8^ Ω·m. The details of the experimental conditions are listed in Table 1. It can be noted that the contact force between two matched AgSnO_2_ contacts is always lower than 5 N in practical low-current-switching devices. Thus, the final load force in the series of experiments was set to 5 N to simulate real working conditions.

## 4. Results and Discussion

### 4.1. Description of the Typical R_c_-F Curve

**Figure 6:**
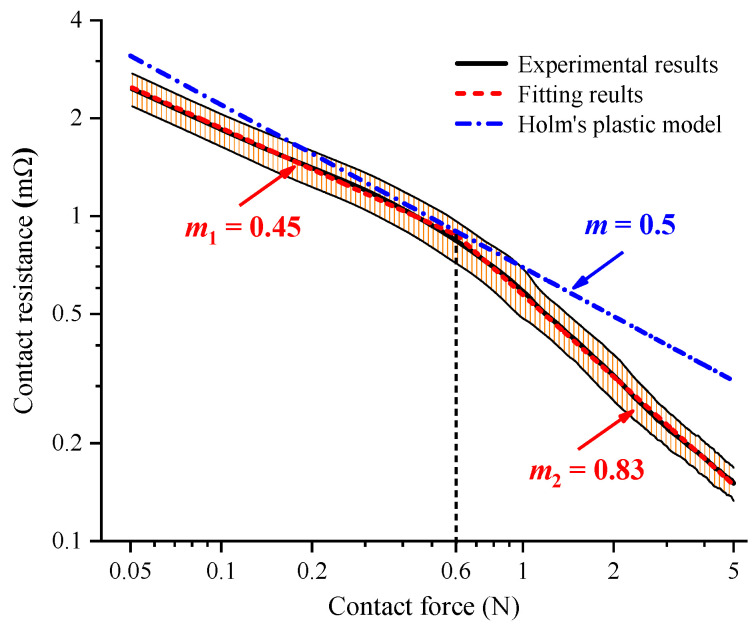
The variation in contact resistance as a function of the load force for AgSnO_2_(12) contacts with bright surfaces.

The variation in the average value and error band of the measured contact resistance of the bright AgSnO_2_(12) electrode material as a function of the load force for an electrical current of 10mA is plotted in Figure 6. The contact resistance curve was calculated statistically from 20 group tests on different electrode samples. The average value of the contact resistance decreased nonlinearly from 2.61 mΩ to 0.19 mΩ when the load force increased from 0.05 N to 5 N. The contact resistance was divided into two different stages and fitted by the power law *R*_c_~*F*_c_^−m^ in the logarithmic coordinates. The fitting results showed that the index *m* was 0.45 when the load force was lower than 0.6 N. After that, the index *m* increased to 0.83, which meant a faster variation rate for contact resistance. Thus, the load force of 0.6 N between two stages was further defined as the transition force *F*_t_. Additionally, the error band in the experimental results indicated that the contact resistance was scattered, even for the same batch of electrode contacts. The fluctuation value of the contact resistance decreased sharply from 0.283 mΩ to 0.124 mΩ when the load force increased from 0.05 N to 0.6 N. Then, this value gently decreased to 0.031 mΩ with the load force of 2.75 N. The fluctuation range further smoothly decreased to 0.018 mΩ, and tended toward stability. The variation in the fluctuation value of the contact resistance by stages indicated that a larger load force was beneficial for the stability of the contact resistance, especially when the load force was larger than 0.6 N. The 0.566 mΩ bandwidth value for the contact resistance error band accounted for 22.98% of the average value of 2.46 mΩ for the load force of 0.05 N. The percentage increased to 23.53% when the load force rose up to 5 N.

The Holm solution for contact resistance for smooth surfaces with plastic deformation calculated with Equation (5) is also plotted in Figure 6 for comparison. It can be noted that the theoretical value of the contact resistance invariably decreased with an exponential power of −0.5 as the contact load force increased from 0.05 N to 5 N. When the load force was lower than the transition force *F*_t_ = 0.6 N, the contact resistance calculation results for the AgSnO_2_(12) electrode material were always higher than the experimental results, and the maximum deviation reached up to 0.65 mΩ when the load force was 0.05 N. After that, the deviation between the calculation and experimental results for the contact resistance decreased to 0.047 mΩ with the load force of 0.45 N. The index *m* of the experimental results gradually approached the theoretical value of 0.5. This could be attributed to the fact that the contact material deformed elastically under a lower load force. When the load force was larger than the transition force of *F*_t_ = 0.6 N, the calculation results for the contact resistance were always higher than the experimental results. The deviation increased slightly and tended to be 0.16 mΩ when the load force rose up to 5 N. The comparison results indicate that the trend towards a decrease in the experimental results with the two studied indexes was different from the contact resistance calculation results obtained with the traditional Holm theory.

### 4.2. Effects of Contact a-Spots on Contact Resistance

**Figure 7:**
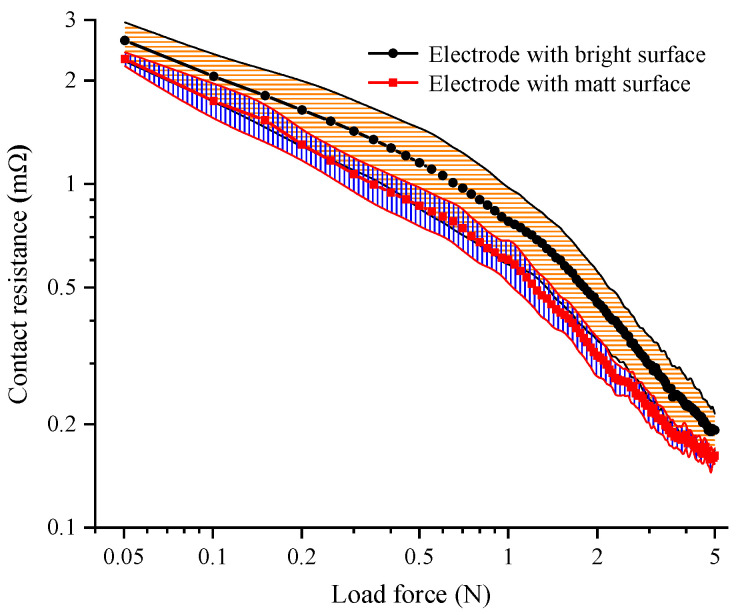
The variations in contact resistance as functions of the load force for AgSnO_2_(12) electrodes with bright and matt surfaces.

The variations in the average values and error bands of the measured contact resistances for the bright and matt AgSnO_2_(12) electrodes as functions of the load force for an electrical current of 10 mA are plotted for comparison in Figure 7. Each contact resistance curve was calculated statistically from 20 group tests on different electrode samples with bright or matt surfaces. As can be seen, the average value of the contact resistance for the matt electrode material was always lower than that of the bright one. The average value and standard deviation for the contact resistance of samples with bright and matt surfaces were 0.192 ± 0.098 mΩ and 0.162 ± 0.008 mΩ for a load force of 5 N, respectively. The contact resistance of the matt electrode decreased by 18.52% compared with that of the bright electrode, which indicates that the matt electrode surface could effectively produce low and stable contact resistance when the load force was set.

**Figure 8:**
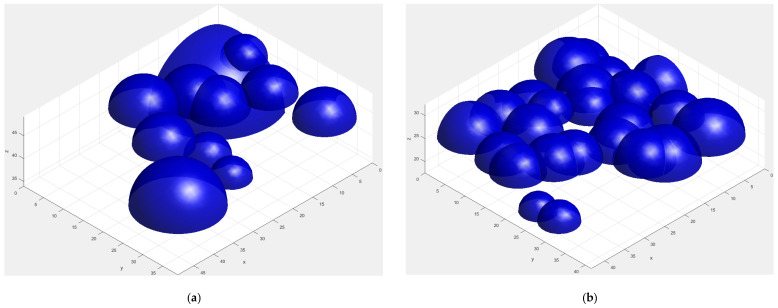
Three-dimensional distributions of the asperities identified with the 9PSP method. (**a**) Bright surface. (**b**) Matt surface.

In order to reveal the influence of electrode surface topography on the contact resistance, the 9-point sphere-peak (9PSP) method was employed to virtually reconstruct the electrode surfaces from spherical asperities. This method can determine spherical asperities in 3D accurately by avoiding the identification of false asperity peaks, such as the saddle point and ridge point, and has been proved with a series of actual engineering surfaces with a broad range of surface roughness [27,28,29]. The identification results showing the asperity numbers and average curvature radii for the bright and matt electrode materials in a scanning area of 1280 μm × 1280 μm are listed in Table 2. The asperity number of the matt surface was 5867, which was more 56.3% than the number of 3754 for the bright surface. The average radius of the electrode with a matt surface was also smaller. The relative positions and height distributions of the identified 3D asperity peaks for the electrodes with bright and matt surfaces are shown in Figure 8. It can be noted that the asperities of the matt surface were denser than those of the bright surface. It can be inferred that there were more contact *a*-spots for the matt electrode in the actual contact process.

For one single circular *a*-spot, in accordance with Equation (3), the produced contact resistance can also be calculated by the contact area *S* as follows:

(6) $$R_{c}=\frac{\rho \sqrt{\pi }}{2\sqrt{S}}$$

Then, it can be assumed that the total area of *N* contact *a*-spots is *S*. Thus, the parallel contact resistance of *N* contact *a*-spots can be written as:

(7) $${R_{c}}^{\prime }=\frac{\rho \sqrt{\pi }}{2\sum_{i=1}^{N}\sqrt{S_{i}}}$$

where *ρ* is the electrical resistivity of the electrode material. The normalized contact resistance *R*_c_^*^ calculated by the contact area of *S* and *S_i_* is as follows:

(8) $$R_{c}{}^{*}=\frac{{R_{c}}^{\prime }}{R_{c}}=\frac{\sqrt{S}}{\sum_{i=1}^{N}\sqrt{S_{i}}}$$

Thus, the normalized contact resistance can be further expressed by the load force *F_i_* for each contact *a*-spot as follows:

(9) $$R_{c}{}^{*}=\frac{\sqrt{F}}{\sum_{i=1}^{N}\sqrt{F_{i}}}$$

**Figure 9:**
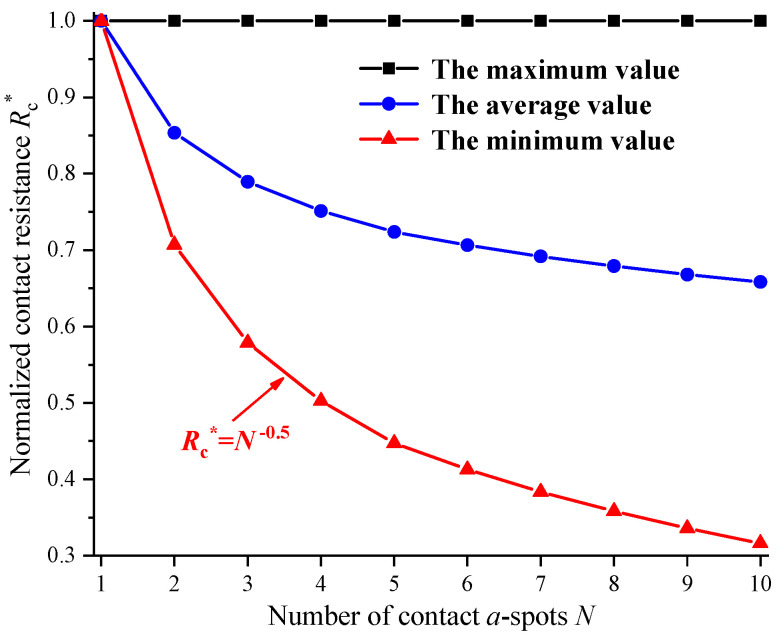
The variations in the minimum, average and maximum normalized contact resistance as a function of the number of contact *a*-spots.

**Figure 10:**
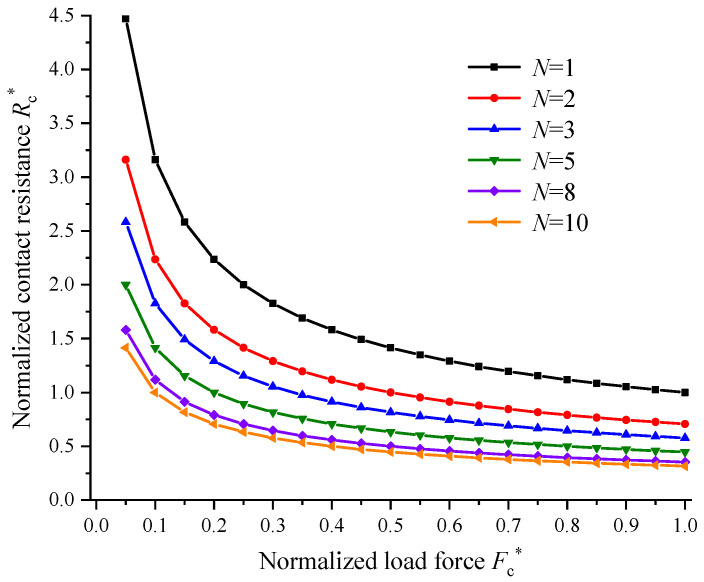
The variations in the normalized contact resistance as a function of the normalized load force with a varying number of contact *a*-spots.

As can be seen, the normalized contact resistance depends closely on the contact area or load force of each contact *a*-spot for the same total contact area or load force. For the case of *N* = 2, there are two contact *a*-spots and the contact areas are defined as *S*_1_ and *S*_2_, respectively. It can be assumed that the initial contact areas of the two contact *a*-spots are *S*_1_ = 0.01*S* and *S*_2_ = *S* – *S*_1_ = 0.99*S*. Then, the contact area *S*_1_ increases in steps of 0.01*S* until *S*_1_ reaches up to 0.5*S*. Thus, the normalized contact resistance with various combinations of contact area for two contact *a*-spots could be calculated with Equation (8). Similarly, the normalized contact resistance when the number of contact *a*-spots increases from *N* = 3 to *N* = 10 can also be calculated. The variations in the minimum, average and maximum normalized contact resistance *R*_c_^*^ as a function of the number of contact *a*-spots were further extracted and are plotted in Figure 9. The variations in the normalized contact resistance *R*_c_^*^ with the same area for each *a*-spot calculated by Equation (9) as a function of the normalized load force F^*^ with a varying number of contact *a*-spots are plotted in Figure 10.

When the area of one contact *a*-spot approaches *S* for *N* contact *a*-spots, the contact resistance is mainly determined by this contact *a*-spot and reaches the maximum value. Moreover, it was found that the average and minimum values of the normalized contact resistance decreased nonlinearly when the number of contact *a*-spots increased. When the number of contact *a*-spots was smaller than 5, the average value of the normalized contact resistance decreased sharply (as shown in Figure 9). The contact resistance always showed an obvious decrement when the number of contact *a*-spots increased one by one (as shown in Figure 10). After that, the variation rate for the normalized contact resistance became slower for the same load force. In addition, the total contact resistance reached the minimum value and always decreased rapidly with an exponential power of −0.5 as the number of contact *a*-spots increased when the area of each *a*-spot was kept equal. This means that the contact resistance was determined by the number of contact *a*-spots for a fixed contact area when the number of contact *a*-spots was relatively smaller. The contact resistance could further drop to the minimum value when the contact area was distributed equally across each contact *a*-spot.

In combination with the contact resistance calculation results (as shown in Figure 9), the average value of the contact resistance was determined by the number of contact *a*-spots. According to the comparative analysis of the surface topography for the electrode materials, the matt surface had more asperities with the given contact region. The contact interface of the matched matte electrode materials could produce more contact *a*-spots under the applied load force. Thus, the average value of the contact resistance for the electrode material with a matt surface was always lower than that for the bright surface when the contact load force increased from 0.05 N to 5 N.

It can be noted that there was an overlapping region in the error band of the contact resistance for the bright and matt surfaces when the load force was less than 2.1 N. This means that the maximum value of the measured contact resistance for the matt surface was larger than the minimum value for the bright surface. On the one hand, the number of contact *a*-spots produced was limited under the lower load force, and the discrete value of the contact resistance was determined by the size of the individual contact *a*-spot. On the other hand, the larger number of newly produced contact *a*-spots accounted for a significant portion of the total number of the existing contact *a*-spots as the contact area extended, which also enhanced the complexity of the sizes of the contact *a*-spots. Therefore, as a joint result of the above factors, the fluctuation in the contact resistance was greater. Furthermore, the individual test values for contact resistance were mostly determined by the size characteristics of the contact *a*-spots and showed greater randomness.

When the load force was larger than 2.1 N, the maximum value of the contact resistance for batches of the electrode contacts with matt surfaces was lower than the minimum value of the contact resistance for the bright surface. It can be inferred that the number of the existing contact *a*-spot was large enough that the newly produced contact *a*-spots had little influence on the contact resistance fluctuation. Thus, the fluctuation ranges in the contact resistances for the two kinds of electrode surface both became narrower, and the increased number of *a*-spots was the main cause of the reduction in the contact resistance. The electrode material with a matt surface, statistically had more *a*-spots and relatively uniformly smaller contact radii, could produce a lower and more stable contact resistance compared to the bright surface with increment in the contact load force.

The above analysis indicates that the number of the contact *a*-spots was the critical factor for the average value of the contact resistance for batches of the electrode contacts, and more contact *a*-spots could produce a lower contact resistance. In addition, the uniform size of the contact *a*-spots was beneficial in restraining the fluctuation and improving the stability of the contact resistance.

### 4.3. Corrected Holm Model

Considering the dispersion of the contact resistance for the batches of electrode contacts, the calculation model for the contact resistance should include average values and fluctuation values in accordance with the measurement results. Thus, the corrected value for the contact resistance $\tilde{R_{c}}$ can be expressed by the calculation value *R*_c_ and error value Δ*R*_c_:

(10) $$\tilde{R_{c}}=R_{c}\pm \Delta R_{c}$$

According to the above analysis, the average value of the contact resistance decreased linearly with two different indexes in the logarithmic coordinates, as shown in Figure 6 and Figure 7. Thus, it could be assumed that the relationship between the calculation value *R*_c_ and load force *F* could be further written as:

(11) $$R_{c}=\left\{\begin{array}{l} K_{1}F^{-m_{1}},\;F_{0}\leq F\leq F_{t}\\ K_{2}F^{-m_{2}},F_{t}<F\leq 5N \end{array}\right.$$

where *F*_0_ = 0.05 N is the initial load force, and *m*_1_ and *m*_2_ are the variation rate of the contact resistance.

It can be noted that the error value for the contact resistance Δ*R*_c_ was closely related to the load force *F* and calculation value of the contact resistance *R*_c_. Thus, it was assumed that the relationship between the error value for the contact resistance Δ*R*_c_ and calculation value of the contact resistance *R*_c_ and the load force *F* was as follows:

(12) $$\Delta R_{c}=\left(k\times F+b\right)\times R_{c}$$

The undetermined parameters *K*_1_, *F*_t_, *m*_1_, *m*_2_, *k* and *b* were obtained by fitting the experimental results for the contact resistance for electrode materials (as shown in Figure 7). The parameter *K*_2_ could be further calculated from *K*_1_ and *m*_1_. The fitting parameters of *F*_t_, *m*_1_, *m*_2_, *K*_1_, *k* and *b* for AgSnO_2_(12) contacts with bright and matt surfaces are shown in Table 3.

**Figure 11:**
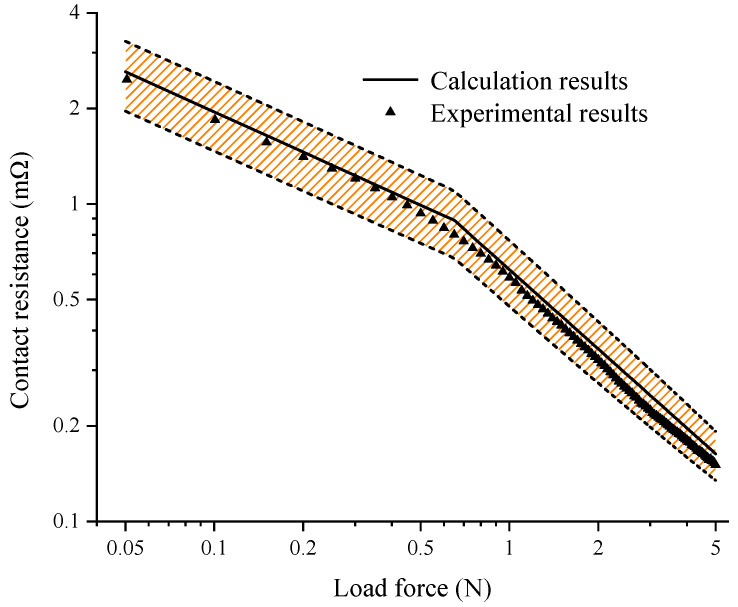
Comparison of contact resistance calculation and experimental results.

The fitting coefficient *b* of the bright surface was about twice that of the matt surface. This meant that the fluctuation range of the contact resistance for the bright surface was larger. A new AgSnO_2_(12) contact with a bright surface, which was excluded from the same batch of the experimental samples used to build the calculation model, was selected to verify the accuracy of the presented corrected model of contact resistance. The variations in the calculation results for the contact resistance with the error band using Equations (11) and (12) and the experimental results as a function of the load force are plotted in Figure 11. The maximum deviation between the average value of the contact resistance and the experimental results was only 4.5% with the load force of 0.6 N. The comparison results indicated that the calculation results for the contact resistance were always in agreement with the experimental results.

## 5. Conclusions

This paper investigated the influences of the surface topography of AgSnO_2_(12) contacts on the relationship between contact resistance and load force using an experimental method and theoretical analysis. Experimental results showed that the contact resistance of the matt electrode decreased by 18.52% compared to that of the bright electrode, which meant that the matt electrode surface could effectively produce low and stable contact resistance when the load force was set. For AgSnO_2_ contacts with rougher surfaces, especially when the root mean square was larger than 27 μm, the number of the contact *a*-spots was the critical factor for the average value of the contact resistance in the batches of the electrode contacts, and more contact *a*-spots could produce lower contact resistance for a given load force. In addition, the uniform size of the contact *a*-spots and the lager load force were beneficial in restraining the fluctuation and improving the stability of the contact resistance. Furthermore, a corrected model of the contact resistance with the error band was produced for AgSnO_2_(12) contacts with matt and bright surfaces. The comparison results proved that the experimental results were always located in the error band of the calculation results. In future, other silver-based electrode materials will be used to establish a general corrected model of contact resistance.

## Figures and Tables

**Table 1:** Experimental conditions.

Parameter	Value
Initial load force	0.05 N
Increment step of load force	0.05 N
Final load force	5 N
Load voltage	6 V
Load current	10 mA
Environment	Temperature: 19–22 °C Humidity: 57% RH

**Table 2:** The statistical parameters of the asperities for electrodes with bright and matt surfaces.

	Number of Asperities (1280 × 1280 μm^2^)	Average Curvature Radius of Asperities (μm)
Bright surface	3754	7.07
Matt surface	5867	6.04

**Table 3:** Fitting coefficients of electrode materials with bright and matt surfaces.

Coefficients	AgSnO_2_(12) with Bright Surface	AgSnO_2_(12) with Matt Surface
*F*_t_	0.6	
*m*_1_	0.45	
*m*_2_	0.83	
*K*_1_	0.866	0.645
*k*	−1.46 × 10^−4^	−1.38 × 10^−4^
*b*	0.249	0.137

## Data Availability

No new data were created or analyzed in this study. Data sharing is not applicable to this article.
